# Effects of DHU001, a Mixed Herbal Formula on Acute Inflammation in Mice

**DOI:** 10.5487/TR.2008.24.3.189

**Published:** 2008-09-01

**Authors:** Young-Doo Back, Hyeung-Sik Lee, Sae-Kwang Ku

**Affiliations:** 15 Department of Herbal Biotechnology, Daegu Haany University, Gyeongsan, 712-715 Korea; 25 Department Anatomy and Histology, College of Oriental Medicine, Daegu Haany University, 290, Yugok-dong, Gyeongsan-si, Gyeongsangbuk-do, 712-715 Korea

**Keywords:** Mixed herbal formula, DHU001, Acute inflammation, Histology, Mouse

## Abstract

The effects of DHU001, a mixed herbal formula consisted of 7 types aqueous extracts for treating respiratory disorders were observed on xylene-induced acute inflammation. The xylene was topically applied 60 min after administration of 500, 250 and 125 mg/kg of DHU001, and all animals were sacrificed 2 hrs after xylene application. The changes on ear weights, histolopathological analyses of ear were evaluated and compared to those of indomethacin and dexamethasone (15 mg/kg treated) - Both of drugs are well-known by anti-inflammatory agents. Xylene application resulted in marked increases in induced ear weights as compared with intact control ear. Severe vasodilation, edematous changes of ear skin and increase in the thickness of the ear tissues, neutrophil infiltration as acute inflammation were detected in xylene-treated control ears at histopathological observation. However, these xylene-induced acute inflammatory changes were dose-dependently decreased by oral treatment of DHU001. Therefore, it is concluded that DHU001 has favorable anti-inflammatory effects on xylene-applicated acute ear inflamed mice.

## INTRODUCTION

Inflammation is an essential protective process preserving the integrity of organisms against physical, chemical and infective insults. However, the inflammatory response to several insults frequently leads to erroneous damage to normal tissues (Habashy *et al.*, 2005). Physical damage, chemical substances, microorganisms and other agents are all possible causes of acute inflammation. The inflammatory responses to such insults consist of changes in blood flow, increased permeability of blood vessels and the subsequent escape of cells from the blood into the tissues. The changes are essentially the same regardless of the cause or its location. Acute inflammation is short-lived, typically lasting only a few days. If the inflammation is longer lasting, however, it is referred to as chronic inflammation. The xylene-induced acute inflammatory mouse ear model has generally been used as one of the classic methods for detecting the efficacy of anti-inflammatory agents. In this model, the anti-inflammatory effect of a drug is based on observations of ear weight and histopathology (Hosseinzadeh *et al.,* 2003; Kou *et al.*, 2005). As control anti-inflammatory drugs, indomethacin are cyclo-oxygenase inhibitors and dexamethasone is a well-known glucocorticoid; these drugs are the most widely used as controls in the development of the new anti-inflammatory drugs (Hosseinzadeh and Younsei 2002; Ramprasath *et al.*, 2006).

DHU001 is a mixed herbal formula consisted of 7 types aqueous extracts; Ficis fructus, Liriopis tuber, Platycodi radix, Schisandrae fructus, Glycyrrhizae radix, Zingiberis rhizome and Menthae herba and being developed for respiratory disorders. Among 7 types of herbal components of DHU001, Liriopis tuber (Park and Geon, 2003), Platycodi radix (Kim *et al.*, 2004), Schisandrae fructus (Narimanian *et al.*, 2005; Rhyu *et al.*, 2006), Glycyrrhizae radix (Sun and Pan, 2006), Zingiberis rhizome (Aimbire *et al.*, 2007; Ghayur *et al.*, 2008) and Menthae herba (Shin, 2003) has been used for treating various respiratory symptoms. In addition, anti-inflammatory effects of Platycodi radix (Ahn *et al.*, 2005; Jang *et al.*, 2006; Kim *et al.*, 2006), Glycyrrhizae radix (Park *et al.*, 2005), Zingiberis rhizome (Ojewole, 2006; Aimbire *et al.*, 2007; Minghetti *et al.*, 2007). In the present study, the effects of DHU001 on the xylene-induced acute inflammation were investigated.

## MATERIALS AND METHODS

**Animals and husbandry.** Sixty-three male ICR mice (6-week-old upon receipt; SLC, Japan) were used after acclimatization for 7 days. Animals were housed five or four per polycarbonate cage in a temperature (20~25°C)- and humidity (40~45%)-controlled room with a 12 h : 12 h light:dark cycle. Feed (Samyang, Korea) and water were supplied *ad libitum*. All animals were fasted overnight before dosing and sacrifice (about 18 h with *ad libitum* access to water).

**Test articles, grouping and dosing.** The herbal compositions of DHU001 were listed in Table 1. Each herbal component was purchased from Cho-Heung Pharmaceutical Ind. Co. (Daegu, Korea) after confirmation of the morphology under microscopy. Approximated amounts of each herbal component was mixed (317.5 g) and boiled in 2 l of distilled water for 2 hours and than filtrated. The filtrate was decompressed using a rotary vacuum evaporator (Lab. Camp, Daejeon, Korea) and lyophilized in a programmable freeze-dryer (IlShin Lab, Daejeon, Korea). Total acquired lyophilized extracts (DHU001) were 34.93 g (yield 11%). Powders of extracts were stored in a desiccator to protect against light and moisture. Indomethacin (Fluka, Switzerland) and dexamethasone (Sigma, USA) were used as reference drugs in this study. The animals were distributed into seven groups with 9 mice per group: intact control, xylene-treated control, 500, 250 and 125 mg/kg DHU001 dosing groups, 15 mg/kg indomethacin and dexamethasone dosing groups. DHU001 was orally administered once, and indomethacin and dexamethasone were administered intraperitoneally once. DHU001 was dissolved in distilled water, and indomethacin and dexamethasone were dissolved or suspended in saline.

**Induction of acute inflammation.** 1 h after dosing with test substances, 0.03 ml of xylene (Merck, Germany) was topically applied to the anterior surface of the right ear. The left ear was considered a control.

**Ear weight measurement.** Two hours after xylene application, all animals were sacrificed and both ears were removed. Circular sections were taken using a cork borer with a 7-mm diameter and weighed as previously described (Kou *et al.*, 2005). The weight of the induced ear was regarded as the absolute weight. The differences between the intact ear and induced ear were calculated as follows:

Differences in weights (g)
= xylene-treated ear weight - intact ear weight

**Histology and histomorphometry.** Following removal, induced ears were trimmed crosswise to include ear cartilage and fixed in 10% neutral buffered formalin, then embedded in paraffin, sectioned (3~4 μm) and stained with Hematoxylin & Eosin stain. The histological profiles of the ears were observed compared to that of intact and/or xylene-treated control. The thickness of the induced ear was calculated using automated image analysis (DMI-300; DMI, Korea) under 50 × magnification (Nikon, Japan). The thicknesses from epidermis to dermis of induced ear skin (anterior skin thickness; μm) and anterior to posterior ear (full ear thickness, μm) were detected as histomorphometry on crossly trimmed ear specimens in regular corresponding regions in all induced ear samples. In addition, infiltrated neutrophil numbers were also calculated as cells/1 mm$^2$ of histological filed.

**Table 1.** Herbal composition of DHU001 used in this study

| Herbs | Scientific name | Amounts (g) |
|---|---|---|
| Ficis fructus | *Ficus carica* Linn. | 140 |
| Liriopis tuber | *Liriope spicata* Lour. | 45 |
| Platycodi radix | *Platycodon grandiflorum* Jacq. | 60 |
| Schisandrae fructus | *Schisandra chinensis* Baill | 22.5 |
| Glycyrrhizae radix | *Glycyrrhiza uralensis* Fisch | 15 |
| Zingiberis rhizoma recens | *Zingiber officinale* Roscoe | 15 |
| Menthae Herba | *Mentha arvensis* Linne var piperascens | 20 |
| Total | 7 types | 317.5 |

All herbs were purchase from from Cho-Heung Pharmaceutical Ind. Co. (Daegu, Korea) and yield 11% aqueous extracts were acquired.

**Statistical analyses.** Multiple comparison tests of the different dose groups were carried out. The variance homogeneity was examined using the Levene test. If the Levene test indicated no significant deviations from the variance homogeneity, obtained data was analyzed using a one way ANOVA test followed by a least-significant difference multi-comparison test to determine which pairs of group comparisons were significantly different. A non-parametric comparison test, Kruskal-Wallis H test was performed in the case of significant deviations from the variance homogeneity was observed at Levene test. When a significant difference was observed in the Kruskal-Wallis H test, the Mann-Whitney U-Wilcoxon Rank Sum W test was used to determine the specific pairs of group comparisons that were significantly different. The SPSS statistical software (Release 6.1.3, SPSS Inc., USA) was used and a $p$ value <0.05 was considered significant.

## RESULTS

**Changes in ear weights.** Significant ($p < 0.01$) increases in the weight of the induced ear were detected in xylene-treated control as compared with intact control and, accordingly, the differences between intact and induced ear were also significantly ($p < 0.01$) increased. However, the induced ear weights and the differences between intact and induced ear of all dosing groups were significantly ($p < 0.01$ or $p < 0.05$) decreased compared to that of xylene-treated control (Table 2).

**Histological analyses of induced ear.** The classic histological profile of acute inflammation, that is, severe vasodilation and edematous changes of skin, was detected in xylene-treated control. However, these histological indicators of acute inflammation were dramatically decreased in all dosing groups as compared with xylene-treated control. In addition, dose-dependency was also demonstrated in the DHU001-treated groups (Fig. 1). Significant ($p < 0.01$) increases in the infiltrated neutrophils, thickness of induced ear (both anterior skin and full ear thicknesses) were detected in xylene-treated control compared to that of intact control. However, these increases in the infiltrated neutrophils and thickness of the induced ear were significantly ($p < 0.01$ or $p < 0.05$) decreased in all dosing groups compared to that of xylene-treated control (Table 3).

## DISCUSSION

The xylene-induced acute inflammation of the mouse ear has generally been used as one of the classic methods for detecting the efficacy of anti-inflammatory agents (Hosseinzadeh *et al*., 2003; Kou *et al*., 2005). In the present study, the effects of DHU001, a mixed herbal formula consisted of 7 types aqueous extracts, on xylene-induced acute inflammation were observed. As the result of pretreatment with DHU001, the changes associated with acute inflammation such as the marked increases of induced ear weight, vasodilation, edematous changes in the skin and increases in the thickness of ear tissues were significantly and dose-dependently decreased. These results are considered direct evidence that DHU001 inhibits the induction of the acute inflammatory response. Previously reported immunomodulatory effects of Platycodi radix (Yoon *et al*., 2003), Glycyrrhizae radix (Sun and Pan, 2006) and Zingiberis rhizome (Tan and Vanitha, 2004), and antioxidative effects of Ficis fructus (Pèrez *et al*., 2003), Liriopis tuber (Han *et al*., 1981), Platycodi radix (Lee *et al*., 2004; Wang *et al*., 2004), Schisandrae fructus (Ko and Lam, 2002), Glycyrrhizae radix (Zhou and Xu, 1992),

**Table 2.** Changes in ear weights in intact or xylene-applicated mice

| Group | Ear weight (g) | | |
|---|---|---|---|
| | Intact ear | Induced ear | Difference |
| Controls | | | |
| Intact | 0.115 ± 0.009 | 0.117 ± 0.006 | 0.002 ± 0.006 |
| Xylene | 0.115 ± 0.009 | 0.151 ± 0.011* | 0.036 ± 0.017* |
| References | | | |
| Dexamethasone | 0.115 ± 0.013 | 0.128 ± 0.019# | 0.013 ± 0.017# |
| Indomethacin | 0.114 ± 0.007 | 0.133 ± 0.009*, ## | 0.018 ± 0.010**,## |
| DHU001 | | | |
| 500 mg/kg | 0.115 ± 0.007 | 0.133 ± 0.017**,# | 0.018 ± 0.017**,# |
| 250 mg/kg | 0.114 ± 0.012 | 0.133 ± 0.017**,# | 0.019 ± 0.012*,# |
| 125 mg/kg | 0.115 ± 0.011 | 0.134 ± 0.012*,## | 0.019 ± 0.018*,# |

Mean ± S.D. of nine mice; Differences = induced ear weight - intact ear weight; *$p < 0.01$ and **$p < 0.05$ compared with intact control; #$p < 0.01$ and ##$p < 0.05$ compared with xylene control.

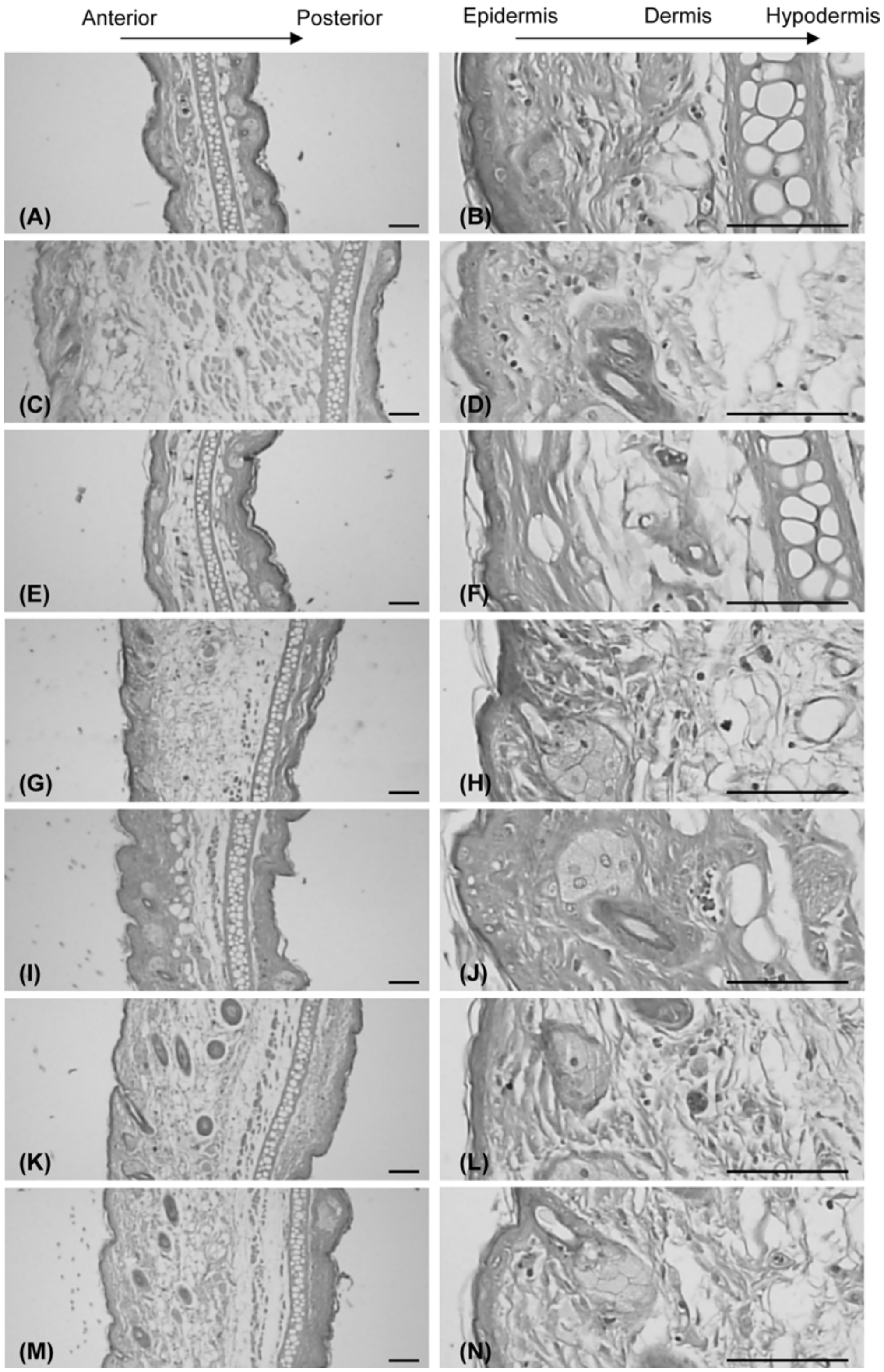

**Fig. 1.** Histological profiles of induced ear detected in intact control (A, B), xylene control (C, D), dexamethasone (E, F), indomethacin (G, H), 500 (I, J), 250 (K, L) and 125 (M, N) of DHU001-administered groups. Note the classic histological profile of acute inflammation as severe vasodilation and edematous changes of skin were detected in xylene control. However, these inflammation indicators were dramatically decreased in all dosing groups tested. All Hematoxylin & Eosin staining, Scale bars = 80 μm.

**Table 3.** The histomorphometric analyses

| Group | Neutrophil numbers (cells/1 $mm^2$ of field) | Thickness of ear (μm) | |
|---|---|---|---|
| | | Full ear thickness | Anterior skin thickness |
| Controls | | | |
| Intact | 8.89 ± 4.17 | 766.765 ± 127.712 | 324.387 ± 94.258 |
| Xylene | 75.56 ± 11.58* | 1916.069 ± 147.049* | 1495.320 ± 166.733* |
| References | | | |
| Dexamethasone | 7.78 ± 5.07$^{\#}$ | 763.837 ± 103.992$^{\#}$ | 328.823 ± 71.952$^{\#}$ |
| Indomethacin | 38.33 ± 12.75$^{*,\#}$ | 1059.186 ± 221.111$^{*,\#}$ | 739.264 ± 185.304$^{*,\#}$ |
| DHU001 | | | |
| 500 mg/kg | 16.11 ± 8.21$^{**,\#}$ | 925.185 ± 115.760$^{\#}$ | 561.230 ± 155.201$^{*,\#}$ |
| 250 mg/kg | 36.67 ± 12.50$^{*,\#}$ | 1317.571 ± 263.949$^{*,\#}$ | 980.569 ± 271.066$^{*,\#}$ |
| 125 mg/kg | 47.22 ± 10.93$^{*,\#}$ | 1543.921 ± 199.440$^{*,\#}$ | 1282.226 ± 221.894$^{*,\#\#}$ |

Mean ± S.D. of nine mice; Anterior skin thickness = thicknesses from epidermis to dermis of induced ear skin; Full ear thickness = thicknesses from anterior to posterior ear of induced ear, $^*p < 0.01$ and $^{**}p < 0.05$ compared with intact control; $^{\#}p < 0.01$ and $^{\#\#}p < 0.05$ compared with xylene control.

Zingiberis rhizome (Masuda *et al.*, 2004) and Menthae herba (Baliga *et al.*, 2003) were considered one of the major mechanisms of the anti-inflammatory effect of DHU001. In the present study, the anti-inflammatory effect of DHU001 was detected, because immunomodulatory agents can reduce inflammation (Ramprasath *et al.*, 2006) and, furthermore, NO synthase inhibitors can reverse several classic inflammatory symptoms (Amin *et al.*, 1995).

After topical application of xylene, marked increases in ear weight were detected as a result of the acute inflammation response; these increases in ear weight have been used as valuable markers for anti-inflammatory effects (Al-Majed *et al.*, 2003; Ojewole 2005). In the present study, the increases in ear weight were dose-dependently inhibited by DHU001 treatment. This inhibition was considered direct evidence that the DHU001 used in this study has favorable effects on reducing the acute inflammatory response.

Histopathologically, severe vasodilation and edematous changes of skin were detected as signs of acute inflammation after topical application of xylene (De La Puerta *et al.*, 1996; Kou *et al.*, 2003; Rotelli *et al.*, 2003; Kim *et al.*, 2007); as a result of these histopathological changes, the thickness of the ear tissues was also markedly increased. However, these histopathological changes and the thickness of ear tissues were dose-dependently decreased after pretreatment with three different dosages of DHU001 in the present study. This inhibition was again considered direct evidence that the DHU001 used in this study has favorable effects on reducing the acute inflammatory response.

Based on the results, we conclude that the DHU001, a mixed herbal formula has somewhat favorable effect in the reduction of the acute inflammatory response induced by xylene application in mice.
